# Redox regulation of gasotransmission in the vascular system: A focus on angiogenesis

**DOI:** 10.1016/j.freeradbiomed.2017.04.025

**Published:** 2017-07

**Authors:** Rajesh K. Mistry, Alison C. Brewer

**Affiliations:** Cardiovascular Division, James Black Centre, King's College London BHF Centre of Excellence, 125 Coldharbour Lane, London SE5 9NU, UK

**Keywords:** SDF-1, stromal cell-derived factor 1, PAD, peripheral arterial disease, GPx, glutathione peroxidase, TAK1, transforming growth factor-B-activated-kinase 1, PTP1B, protein tyrosine phosphatase 1B, EDRF, endothelial-derived relaxing factor (EDRF), BH_4_, tetrahydrobiopterin, L-NMMA, NG-monomethyl-L-arginine, L-NAME, NG-nitro-L-arginine-methyl ester, HRE, hypoxia response element, ADMA, asymmetric dimethylarginine, DDAHI, dimethylarginine dimethylaminohydrolase I, DDAHII, dimethylarginine dimethylaminohydrolase II, NAC, N-acetylcysteine, CORM-1, tricarbonyl-dichlororuthenium (II), CORM-3, tricarbonylchloro(glucinato)ruthenium (II), CORM, carbon monoxide releasing molecule, SnPPIX, tin protoporphyrin IX, ZnPP, zinc protoporphyrin, Bach-1, BTB Domain and CNC Homolog 1 (Bach1), HNO, nitroxyl, Reactive oxygen species, Nitric oxide, Carbon monoxide, Hydrogen sulphide, Angiogenesis, NADPH oxidase 4

## Abstract

Reactive oxygen species have emerged as key participants in a broad range of physiological and pathophysiological processes, not least within the vascular system. Diverse cellular functions which have been attributed to some of these pro-oxidants within the vasculature include the regulation of blood pressure, neovascularisation and vascular inflammation. We here highlight the emerging roles of the enzymatically-generated reaction oxygen species, O_2_^-^ and H_2_O_2_, in the regulation of the functions of the gaseous signalling molecules: nitric oxide (NO), carbon monoxide (CO), and hydrogen sulphide (H_2_S). These gasotransmitters are produced on demand from distinct enzymatic sources and in recent years it has become apparent that they are capable of mediating a number of homeostatic processes within the cardiovascular system including enhanced vasodilation, angiogenesis, wound healing and improved cardiac function following myocardial infarction. In common with O_2_^-^ and/or H_2_O_2_ they signal by altering the functions of target proteins, either by the covalent modification of thiol groups or by direct binding to metal centres within metalloproteins, most notably haem proteins. The regulation of the enzymes which generate NO, CO and H_2_S have been shown to be influenced at both the transcriptional and post-translational levels by redox-dependent mechanisms, while the activity and bioavailability of the gasotransmitters themselves are also subject to oxidative modification. Within vascular cells, the family of nicotinamide adenine dinucleotide phosphate oxidases (NAPDH oxidases/Noxs) have emerged as functionally significant sources of regulated O_2_^-^ and H_2_O_2_ production and accordingly, direct associations between Nox-generated oxidants and the functions of specific gasotransmitters are beginning to be identified. This review focuses on the current knowledge of the redox-dependent mechanisms which regulate the generation and activity of these gases, with particular reference to their roles in angiogenesis.

## Highlights

• Reactive Oxygen Species and Gasotransmitters are critical regulators of angiogenesis and are involved in processes both upstream and downstream of VEGF expression.
• The enzymes which generate NO, CO and H_2_S are redox regulated at the levels of their transcription, translation, activity and bioavailability.
• NAPDH oxidases are functionally significant sources of superoxide and hydrogen peroxide that can direct the functions of specific gasotransmitters.
• The products of the chemical interactions of reactive oxygen species and the gasotransmitters are emerging as novel biomolecules that may be exploited therapeutically.

## Graphical abstract

**Figure:**
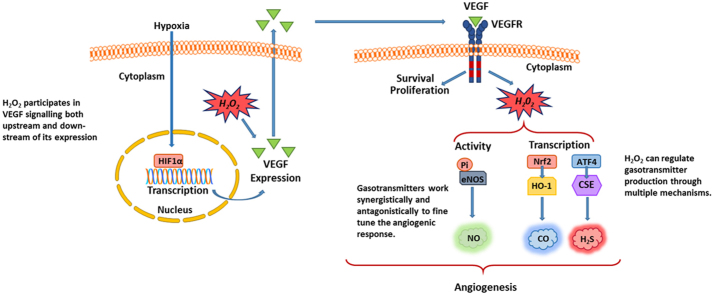

## 1 Introduction

**Fig. 1:**
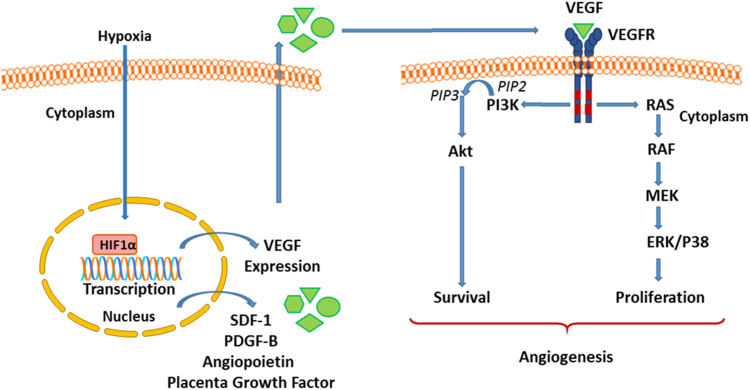
A schematic illustration of hypoxia- and VEGF-mediated signalling in the endothelium leading to angiogenesis through the promotion of cell survival and proliferation. In response to hypoxia, the upregulation of HIF-1α leads to increased expression of a number of pro-angiogenic factors including SDF-1, PDGF-B, angiopoietin, placenta growth factor and importantly VEGF. VEGF signals have been the best characterised and have been shown to cause the stimulation of VEGFR2 within the endothelium. In turn this activates downstream signalling pathways including P13K/Akt and MEK/MAPK to promote pro-angiogenic cellular responses.

The vascular system represents a closed circuit of vessels that collectively transports oxygen and nutrients to all tissues. This delivery system has evolved multiple mechanisms that allow it to adapt to dynamic changes in tissue physiology in order to ensure that the supply of oxygen and nutrients matches the tissue demand at any given time [1]. One way in which this is achieved is through the growth of new blood vessels from the existing vasculature in a multistep, highly coordinated process known as angiogenesis. In this process, activated (“sprouting”) endothelial cells (from either arterial or venous vessels) release proteases which degrade the underlying basement membrane and subsequently migrate into the extracellular matrix. Adjacent endothelial cells in turn proliferate, differentiate and form luminal tubes which eventually fuse to the pre-established vascular network [2]. These adaptive responses are biochemically coordinated by a number of cell signalling mediators, most notably the vascular endothelial growth factor (VEGF) family of protein growth factors [3]. VEGF targets the endothelium to initiate angiogenesis and comprises multiple isoforms of which VEGF-A is a key regulator of vessel growth which has been shown to induce endothelial cell growth and survival both *in vitro* and *in vivo* [4], [5], [6]. The central role of VEGF-A in physiological angiogenesis (and vasculogenesis) has been demonstrated during embryonic and early postnatal development [6] as well as in pathological angiogenesis seen in solid tumour formation [7]. At the biochemical level, the effects of VEGF are mediated *via* its binding to endothelial-expressed, plasma membrane-bound, tyrosine kinase receptors, Flt-1 (VEGFR-1) and primarily, Flk-1/KDR (VEGFR-2). VEGF binding to VEGFR-2 initiates its autophosphorylation, dimerization and the subsequent activation of its tyrosine kinase domain [8]. This in turn activates downstream signalling cascades, including the MEK-ERK1/2 pathway to support cell growth and proliferation [4] as well as the anti-apoptotic phosphoinositide 3-kinase- (PI3-K-)Akt pathway to promote cell survival [5] (Fig. 1).

Increased VEGF-dependent signalling triggers the angiogenic response and therefore the control of VEGF expression is critical to the regulation of angiogenesis. In this regard, the transcriptional regulation of VEGF appears to play the pre-eminent role, and multiple transcription factors which are positive mediators of VEGF transcription have been identified, together with cellular agents which stimulate their activity through diverse signalling pathways [9]. An important stimulus for angiogenesis is tissue hypoxia and, accordingly, VEGF is a known direct transcriptional target of hypoxia-inducible factor 1 (HIF-1). Similarly, the expressions of other known pro-angiogenic factors including angiopoietin 1 and 2, stromal cell-derived factor-1 (SDF-1), placenta growth factor and platelet-derived growth factor B are also known to be upregulated by HIF-1 [10], [11].

These regulatory pathways, both upstream and downstream of the action of VEGF, have been extensively studied and emerging data indicate the involvement of redox-dependent molecular signalling mechanisms at multiple stages [12]. Further, angiogenic responses have increasingly been shown to be mediated in part by the biological actions of a small family of gases, termed “gasotransmitters”, which are enzymatically generated within vascular cells [13]. The precise mechanisms of the regulation of action of these short-lived mediators, which comprise nitric oxide (NO), carbon monoxide (CO) and hydrogen sulphide (H_2_S) are not currently fully understood. However, there is growing evidence that their generation may be regulated in part by redox-dependent mechanisms, while their chemical nature in some cases makes them highly susceptible to oxidation. In this review we summarise the current knowledge of the biochemistry which links reactive oxygen species generation, redox signalling and the action of the gasotransmitters in angiogenesis. A more comprehensive understanding of these mechanisms would be of great potential benefit in identifying new therapeutic targets for both cancer and vascular diseases such as peripheral arterial disease (PAD) [14].

### 1.1 Reactive oxygen species and redox-signalling

**Fig. 2:**
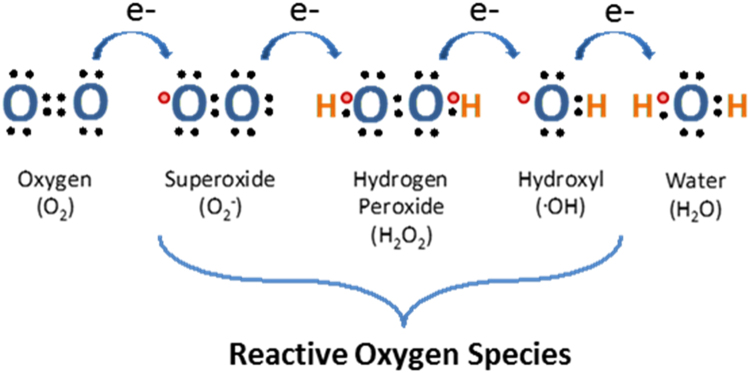
**Reactive oxygen species**. A diagrammatic illustration depicting the gradual reduction of molecular oxygen (O_2_), through its sequential gain of electrons, to form water. As oxygen is reduced various reactive oxygen species are generated in the process including superoxide (O_2_^-^), hydrogen peroxide (H_2_O_2_) and hydroxyl radicals (•OH).

Reactive oxygen species are partial reduction products of molecular oxygen (O_2_) and include superoxide (O_2_^-^), hydrogen peroxide (H_2_O_2_) and the hydroxyl radical (•OH) (Fig. 2). Historically, they have been thought of as merely potentially detrimental by-products of aerobic metabolism in the mitochondria or the result of unregulated uncoupling of various O_2_-dependent enzymatic reactions [15]. The harmful biological effects of these oxidants are countered by the actions of enzymatic and non-enzymatic antioxidants that collectively form the cellular antioxidant system. Enzymatic antioxidants include superoxide dismutase (SOD) that removes O_2_^-^, as well as catalase, peroxiredoxin and glutathione peroxidase (GPx) that metabolise H_2_O_2_ [16]. Non-enzymatic antioxidants include vitamins C and E as well as the major redox buffer, glutathione (GSH). GSH is present at millimolar concentrations in the cell and scavenges both H_2_O_2_ and free radicals through the formation of oxidised GSH (GSSG). The “steady state” cellular redox status is maintained through the balance of constitutively expressed pro-oxidants and these antioxidant systems (reviewed in [17]).

However it is now understood that physiological, enzymatic sources of H_2_O_2_ and O_2_^-^ exist which generate these species in a tightly regulated and localised fashion. Moreover, the short-lived, regulated production of these pro-oxidants can mediate various homeostatic aspects of intracellular and extracellular function [18], [19]. Both O_2_^-^ and H_2_O_2_ are capable of orchestrating a variety of cell signalling responses that culminate in a broad range of phenotypic outcomes such as altered proliferation, adhesion and invasion of endothelial cells. Thus they can act as intracellular second messengers in various signalling pathways to control cell function. Due to differences in the chemical properties of each type of oxidant species, such as half-life and lipid solubility, not all reactive oxygen species are able to function as efficient signalling mediators [20]. For example, O_2_^-^ has a short half-life of only 1 μS and is membrane-impermeable due to its polarity. Therefore its signalling capacity is limited. By contrast, H_2_O_2_ is more stable and has a relatively long half-life of 10 μS. It can freely permeate biological membranes and has emerged as the major redox metabolite mediating redox signalling events [21]·H_2_O_2_ acts to modulate numerous cellular processes *via* diverse mechanisms including the regulation of gene transcription, mRNA and/or protein stability, intracellular trafficking and protein activity [22]. The precise mechanisms which underlie the regulation of the generation of H_2_O_2_ and the specificity of its action are not fully understood. However it is clear that in complex metazoan systems, the modulation of redox-signalling events must include intracellular compartmentalisation and gradients of the oxidative signal, together with differential reactivity of the target sensors and bioavailability of buffering thiol peroxidase systems [21], [22].

**Fig. 3:**
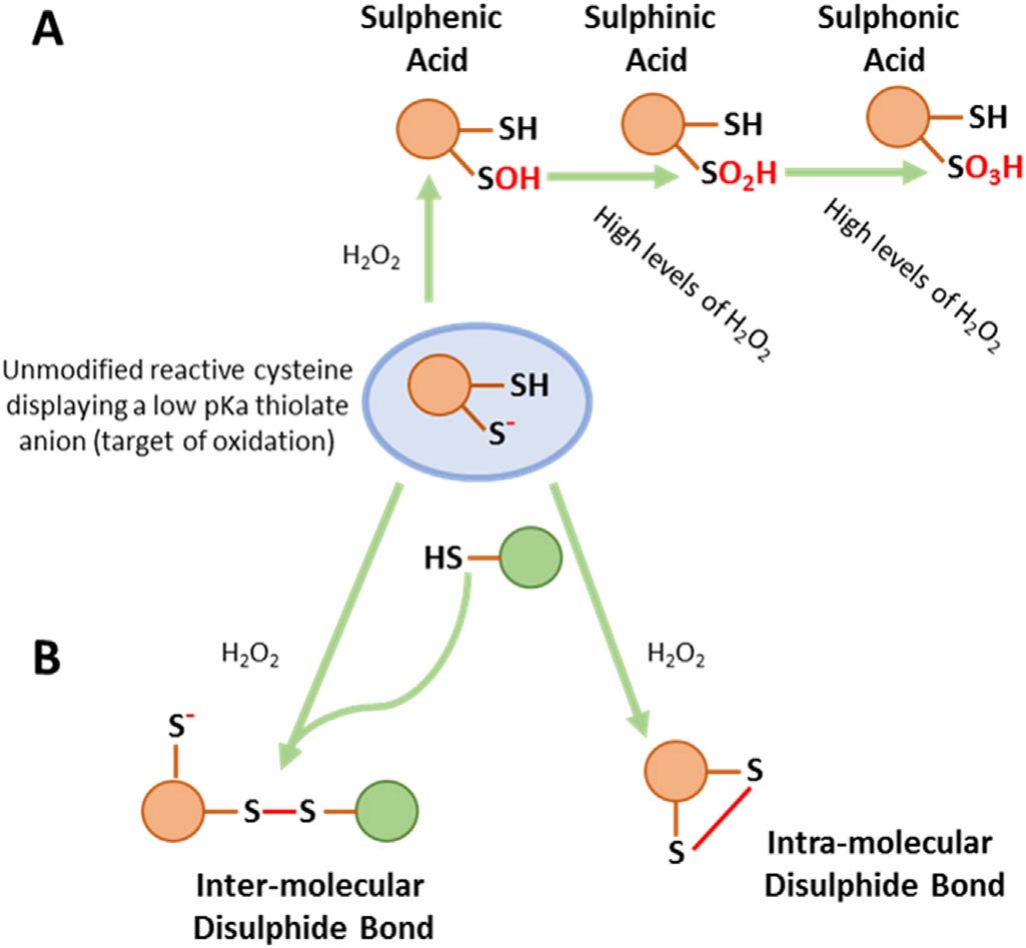
**H_2_O_2_-induced post-translational modifications.** A schematic diagram indicating H_2_O_2_-induced post-translational modifications of a cysteine thiol moiety. The pKa, which is a measure of hydrogen dissociation from a given thiol, is determined by the microenvironment in which the thiol is situated, with basic surrounding amino acids tending to deprotonate the thiol more efficiently than acidic amino acids **A)** The progressive oxidation of a deprotonated, reactive thiol by H_2_O_2_ proceeding through sulphenic, sulphinic and sulphonic acid states. **B)** Inter and Intra-molecular disulphide bond formation. These modifications lead to functional changes within a protein.

Physiological redox signals are transmitted though the reversible oxidative modification of target proteins to alter conformation and function·H_2_O_2_ can act to modify cysteine residues on redox-sensitive proteins [23] that possess reactive thiol groups which exhibit a low pKa [18] (Fig. 3). A number of different types of H_2_O_2_-induced, post-translational modifications exist, including those that involve the sequential oxidation of a thiol which proceeds through sulphenic acid (SOH), sulphinic acid (SO_2_H) and sulphonic acid (SO_3_H) states [24] (Fig. 3). In addition to these modifications, H_2_O_2_ can also induce covalent intra- and inter-disulphide bond formation to elicit a functional change (Fig. 3) [25], [26]. Of these oxidation states, cysteine-sulphenic acids and disulphides are readily reversible *via* the cell's intrinsic glutathione and thioredoxin antioxidant systems, while cysteine-sulphinic acid derivatives, (once thought to be irreversible), can also be reduced the action of sulfiredoxins [27]. By contrast, cysteine sulphonic acid modifications are not known to be reversible. Both O_2_^-^ and H_2_O_2_ can also react with metal centres, often located within the active sites of target proteins. For example, H_2_O_2_ can react with a Mn-Fe centre in the active site of protein phosphatase-1 leading to its inhibition [28].

### 1.2 Functional vascular sources of reactive oxygen species: NADPH oxidases

Within vascular cells, O_2_^-^ and H_2_O_2_ is derived from a number of enzymatic sources including the mitochondrial electron transport system, cytochrome p450, xanthine oxidase and uncoupled nitric oxide synthase (NOS). As stated above, the O_2_^-^ derived from these sources is generally considered to be the result of mis-regulated metabolic functions, although there is some evidence for its potential functional importance in some cellular signalling pathways. By contrast, the family of flavoenzymes known as Nicotinamide Adenine Dinucleotide Phosphate Oxidases (NAPDH oxidases/Noxs) generate O_2_^-^ or, in some cases, H_2_O_2_ in a tightly regulated manner as their sole biological function [29]. The prototype NADPH oxidase, originally identified in phagocytes, comprises the membrane-associated catalytic gp91phox (now termed Nox2) and regulatory p22phox subunits, in addition to the cytosolic subunits; p47phox, p67phox and p40phox together with the small GTPase, Rac1. Nox homologues were subsequently identified in non-phagocytic cells, and in humans, NADPH oxidases now comprise a known family of 7 multi-subunit transmembrane proteins (each containing a distinct catalytic subunit; Nox1-5 or duox 1 and 2) which display distinct cell-type and subcellular expression patterns [29], [30]. Each catalytic Nox subunit contains at least 6 transmembrane alpha helices and, with the exception of Nox5 and duox1/2, all isoforms associate with p22^phox^. However, different Nox isoforms additionally associate with other varied regulatory proteins which modulate their regulation and function. For example, p22^phox^ can associate with Polymerase Delta Interacting Protein 2 [31] to activate Nox4 in smooth muscle cells (SMCs) whereas Nox1 activation is mediated by association with Nox organiser 1 (NOXO1) and Nox activator 1 (NOXA1) [32]. Mechanistically, all the Nox subunits shuttle electrons from NADPH down an electrochemical gradient across a membrane to molecular oxygen (O_2_) which is subsequently reduced to superoxide (O_2_^-^) [33], [34]. However, in the case of some Noxs (such as Nox4 and duox1/2) the O_2_^-^ that is produced is very rapidly converted to H_2_O_2_. The exact mechanism(s) by which this is achieved is still unclear although some studies suggest that Nox4 may be capable of mediating an intrinsic superoxide dismutase activity [35]. O_2_^-^ or H_2_O_2_ newly-generated by Noxs can be utilised by the cell in order to elicit tightly-controlled, cellular responses [29], [30], [36]. Moreover, the differences in structure, activity, expression, regulation and the type of oxidant species produced enable each specific Nox protein to direct its own distinct and defined functions [37], [38], [39].

Nox proteins are expressed in all of the component cell types of the vascular wall. SMCs express Nox1, Nox4 and Nox5 whereas the endothelium contains 4 Nox isoforms; Nox1, Nox2 (including the associated phagocytic subunits), Nox4 and Nox5 [40]. Their distinct physiological (and pathophysiological) roles in vascular function have, in some cases, been demonstrated in mouse models *in vivo*. For instance, genetic ablation of Nox1 or Nox2 was shown to elicit beneficial effects on hypertension and vascular dysfunction in certain pathophysiological settings [41], [42], [43], [44], [45]. By contrast, deficiency of Nox4 in mice acted to promote angiotensin II-dependent vascular dysfunction, suggesting that Nox4-generated H_2_O_2_ plays a protective role in vascular cells [46]. The different functions of Nox2 and Nox4 within the vasculature have been further illustrated by the contrasting phenotypes of transgenic (TG) mice which overexpress these genes specifically within endothelial cells. Thus we demonstrated that endothelial-specific overexpression of Nox2 (directed by the Tie2 promoter) resulted in increased blood pressure in mice after angiotensin II infusion, and attenuated acetylcholine- (Ach-)induced vasorelaxation in isolated aortas [47]. Meanwhile Tie2-mediated overexpression of Nox4 within endothelial cells acted to reduce basal blood pressure within TG mice, and enhanced endothelial-dependent vasodilation *ex-vivo* [48]. Therefore Nox4 is increasingly considered to be a positive regulator of vascular homeostasis [48], [46], [49], and in this regard it may be significant that it is abundantly expressed in the endothelium, compared to other Nox isoforms [39], [50], [51].

### 1.3 Reactive oxygen species and redox signalling in angiogenesis

There is now a large body of evidence which demonstrates that both O_2_^-^ and H_2_O_2_ participate in redox-dependent signalling pathways which can modulate angiogenic responses both *in vitro* and *in vivo*. Early *in vitro* studies demonstrated that topical application of H_2_O_2_ to bovine thoracic aortic endothelial cells (BAECs) enhanced proliferation, migration and tube formation compared to controls and that these effects could be ablated by the administration of catalase [52]. Perhaps significantly, it has been reported that these H_2_O_2_-induced angiogenic responses occur in a biphasic manner, with low concentrations of H_2_O_2_ enhancing angiogenic phenotypes and higher concentrations inhibiting them [53]. In addition, as might be expected from their very diverse properties, there is specificity with regard to the angiogenic effects of individual reactive oxidant species. For example, O_2_^-^ and H_2_O_2_ were shown to be both pro- and anti-angiogenic at varying concentrations, whereas •OH was found only to exert anti-angiogenic effects, or to have no effect at all at low concentrations [53].

Many studies have now shown that some reactive oxygen species can promote the induction of VEGF expression in both endothelial and smooth muscle cells [54], [55]. In addition, VEGF (and other angiogenic growth factors such as angiopoietin-1) have been shown to elicit angiogenic cellular responses *via* O_2_^-^- and/or H_2_O_2_-dependent molecular mechanisms [56]. VEGF-induced endothelial cell sprouting, mediated *via* Transforming growth factor-β-activated-kinase 1 (TAK1), was also shown to involve the increased expression of (mitochondrial-expressed) SOD2. The functional involvement of H_2_O_2_ (generated by SOD2) was demonstrated as the impaired angiogenesis, observed in aortic rings in which endothelial TAK1 was ablated, could be rescued by the overexpression of SOD2 [57]. Thus reactive oxygen species are involved in the regulation of cellular angiogenic responses, both upstream and downstream of the induction of VEGF expression. *In vivo*, a potential role for H_2_O_2_ in angiogenesis was first demonstrated in extra-cellular-SOD-transgenic mice, which displayed increased H_2_O_2_ production and angiogenesis in a murine model of hind-limb ischemia [58]. Moreover, increased H_2_O_2_, VEGF production and Akt phosphorylation, associated with the promotion of physiological pulmonary angiogenesis, was also observed in exercise-trained rats [59].

### 1.4 Vascular NADPH oxidases and angiogenesis

The physiological sources of reactive oxygen species which regulate angiogenic cellular processes are potentially varied, and there is evidence for the involvement of O_2_^-^ and H_2_O_2_ derived from several different sources in some *in vitro* settings. For instance, the stimulation of human retinal endothelial cells with high levels of glucose increased VEGF expression and cell proliferation in a manner dependent on mitochondrial O_2_^-^ production from the electron transport chain (ETC) [60]. Moreover, a recent study demonstrated that treatment of human umbilical vein endothelial cells (HUVECs) with VEGF significantly elevated mitochondrial H_2_O_2_ production and subsequent cell migration [61]. In addition, the inhibition of endogenous xanthine oxidase was shown to reduce VEGF-stimulated Akt phosphorylation in HUVECs suggesting that xanthine oxidase may also be important for VEGF- induced endothelial cell survival [62]. However the major sources of O_2_^-^ and H_2_O_2_ which function as signalling molecules in the angiogenic processes in vascular cells are recognised as the NADPH oxidases [56].

The (patho)physiological importance of Nox1, 2 and 4 in angiogenic processes *in vivo* has, in each case, been demonstrated. Global genetic ablation of Nox1 was shown to impair tumour angiogenesis in mice [63], while (again global) genetic deletion of Nox2 or Nox4 was reported to reduce blood flow recovery in ischemic mouse hindlimb models [46], [64]. Nox4 expression was further shown to be required to support exercise-induced angiogenesis in mice [65]. However, in a recent study in which the consequences of the ablation of Nox1,2 or 4 were assessed in a slow-growing mouse tumour model, only Nox4 was found to contribute positively to angiogenesis. By contrast, ablation of Nox2 had no effect, and deletion of Nox1 actually enhanced angiogenesis [66]. The significance of the apparent discrepancies in these findings is not clear, but likely reflects the different experimental models adopted. The physiological importance specifically of Nox4 in angiogenesis is further evidenced by endothelial-specific TG overexpression of Nox4 in mice which demonstrated increased angiogenesis in an ischemic hind limb model [49]. Accordingly, *in vitro*, Nox4 overexpression was shown to increase endothelial cell proliferation, migration and tube formation [49], [67]. It is perhaps also significant that Nox4 is an inducible gene, whose transcriptional expression is activated by hypoxia [49], [67]. This clearly may have relevance with regard to its potential functional role in the regulation of angiogenesis.

At the molecular level, the mechanisms which underlie the angiogenic functions of Noxs have been extensively studied *in vitro*. Nox-dependent upregulation of HIF1α and VEGF expression *via* Akt and ERK1/2 activation in prostate cancer cells was demonstrated by siRNA-mediated targeting of the common p22 phox subunit [68]. In the specific case of Nox1, overexpression in NIH3T3 fibroblasts promoted VEGF and VEGFR expression in an H_2_O_2_-dependent manner [69]. The (mRNA and protein) expression of Nox1 was also shown to be increased by hypoxia in A549 (epithelial) cells, which in turn resulted in increased HIF expression (the transcriptional activator of VEGF), that could be blocked by catalase, or the flavoprotein inhibitor diphenylene iodonium (DPI). Both Nox1 and Nox2 are activated by the small GTPase, Rac1 [29]. AGS cells (gastric cancer cells) in which Rac1 expression had been depleted showed reduced expression of both HIF-1α and VEGF, while media conditioned by these cells inhibited cell proliferation when applied to human endothelial cells ( [70]). A potential function for Nox4 in the upstream activation of VEGF expression has also been demonstrated, as siRNA-mediated inhibition of Nox4 expression resulted in decreased HIF2-α and VEGF promoter activity in HEK-293 fibroblasts [71]. In addition, overexpression of Nox4 in human microvascular endothelial cells (HMVECs) was sufficient to increase VEGF mRNA levels, while downregulation of Nox4 decreased the level of (HIF-1α-dependent) VEGF expression after insulin stimulation [72].

There are also many reports of the involvements of Noxs in the signalling pathways downstream of VEGF and other pro-angiogenic growth factors. VEGF and angiopoietin-1 have been shown to stimulate endothelial cell migration in a Nox2-dependent manner [73], [74]. In addition, Nox4 was shown to mediate VEGF-dependent angiogenic reponses in human microvascular endothelial cells *in vitro*, by enhancing receptor tyrosine kinase phosphorylation and the activation of ERK1/2 [75]. Moreover in a separate study, VEGF stimulation resulted in a physical interaction between phosphorylated VEGFR2 and Nox4 that promoted STAT3-dependent proliferation of endothelial cells [76]. Finally, a study by Evangelista et al. demonstrated that VEGF induced endothelial migration in a Nox4- and Nox2-dependent manner and that this involved the S-glutathiolation of SERCA2b which subsequently increased Ca^2+^ influx into endothelial cells [77]. The precise protein targets of Nox-generated O_2_^-^ and/or H_2_O_2_ which are susceptible to oxidation and act to modulate the angiogenic responses in endothelial cells are not known. However it has been demonstrated that H_2_O_2_ produced by ectopic expression of extracellular SOD (ecSOD) can increase VEGF-induced phosphorylation of VEGFR2 *via* the oxidative inactivation of protein tyrosine phosphatase 1B (PTP1B) (a negative regulator of VEGFR2 signalling) [58]. It is of note that in many separate studies PTP1B has also been shown to be a target of oxidative modification by Nox4 [78], [79]. In addition to PTP1B, VEGFR2 has also been shown to have redox-sensitive cysteine residues in its kinase domain [80] and it has been suggested that H_2_O_2_ can induce the formation of an inhibitory intramolecular disulphide bond in VEGFR2 [81]. However the functional significance of this with respect to the cellular angiogenic responses to H_2_O_2_ remains to be determined.

A central principle in intracellular redox-signalling mechanisms is the localization of the (freely diffusible) signalling molecules close to their targets. The enzyme complexes generating these signals (such as H_2_O_2_-generating Nox4), must therefore be localised to specific intracellular compartments in order to effect specific responses. The subcellular location of Nox4 has been the subject of some controversy, and has been reported, in disparate cells types, in the nucleus, endoplasmic reticulum (ER), mitochondria, focal adhesions and plasma membrane (reviewed in [82]). The underlying reasons for these discrepancies may reflect cell-type specific differencies in Nox4 function, or the poor specificity of available antibodies. However, in the case of the Nox4-dependent oxidation of PTP1B, (described above), it has been shown that the co-localization of Nox4 and PTP1B within the ER of endothelial cells was a functional requirement [78].

### 1.5 Gasotransmitters

It is now known that in addition to the involvement of reactive oxygen species (highlighted above) another group of endogenously-produced small molecules appear to be important for vascular responses both *in vitro and in vivo,* including angiogenesis, cell survival and the regulation of vascular tone [83], [84], [85]. Collectively termed “gasotransmitters”, NO, CO and H_2_S comprise a group of small molecule signalling agents which were previously known as toxic gases [86] but are now recognised as physiological regulators and effectors in diverse biological processes. They have become the subject of much research and are now recognised to contribute to signalling pathways in angiogenesis both upstream and downstream of VEGF (and other angiogenic growth factor) expression as described below.

The term gasotransmitters is, however, somewhat misleading as these molecules are completely soluble at physiologically-relevant concentration and pH and so perhaps should not be considered to be gases in these circumstances. None-the-less, their discovery and the characterisation of their mechanisms of action has altered the conventional paradigm of intercellular signalling. Unlike conventional signalling molecules (such as peptides) that are often stored in vesicles, gasotransmitters are generated on demand by distinct enzymes. Furthermore, peptide signals tend to mediate their function by binding to plasma membrane receptors such as receptor tyrosine kinases. By contrast, gasotransmitters can diffuse across membranes both within and between cells and subsequently interact with their protein target(s) directly to modify structure and function [84]. In this regard they share similar properties with some reactive oxygen species, most notably H_2_O_2_. Their biochemical targets (in common with both O_2_^-^ and/or H_2_O_2_) are redox metal centres (most notably iron-containing haem proteins) and redox-active amino acids such as cysteine thiol groups. Chemical reactions between the gasotransmitters and some reactive oxygen species (such as the reaction between O_2_^-^ and NO to form peroxynitrite (NOO^-^)), together with significant commonality in their targets, results in a significant interdependence between these groups of signalling molecules in their biological functions [87]. Thus the specific chemical properties and reactivity of the signalling oxidant molecules and the gasotransmitters have been utilised together to modulate complex signalling pathways as is beginning to become apparent.

**Fig. 4:**
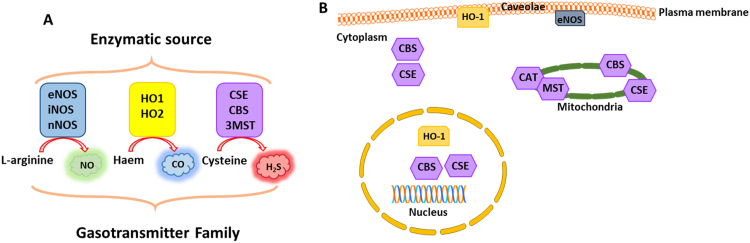
**Gasotransmitters**. A schematic diagram depicting the major gasotransmitters and their respective enzymatic sources and subcellular locations. **A)** The relevant vascular sources of these gases are eNOS for NO, HO-1 for CO and CSE for H_2_S. eNOS: Endothelial Nitric Oxide Synthase, nNOS: Neuronal Nitric Oxide Synthase, iNOS: Inducible Nitric Oxide Synthase, HO-1: Haem oxygenase-1, CSE: Cystathionine-γ-lyase, CBS: Cystathionine-β-synthase, and 3MST: 3 mercaptopyruvate sulphur transferase. **B)** Intracellularly, eNOS is located in caveolae at the plasma membrane, HO-1 can be found in caveolae and the nucleus, CSE and CBS are located in the cytoplasm and may also be found in the nucleus and mitochondria. CAT and MST are located in the mitochondria.

As stated above, gasotransmitters must be synthesised, as required, close to their specific sites of action [84] and therefore both the expression and the activity of their biosynthetic enzymes must be tightly controlled. Within the vascular system, the relevant enzymes involved in the generation of these gases are endothelial nitric oxide synthase (eNOS) for NO [88], haem oxygenase-1 (HO-1) for CO [89] and cystathionine gamma-lyase (CSE) for H_2_S [90] (Fig. 4). In addition to the modulation of function of the gasotransmitters by their direct reactivity (in some cases) with O_2_^-^ and/or H_2_O_2_, there is also increasing evidence that the expression and/or function of each of these biosynthetic enzymes is regulated by redox-dependent mechanisms. Therefore the gasotransmitters should, as a group, be considered to be important effectors of redox-dependent signalling mechanisms which regulate the angiogenic cellular responses, as detailed below.

### 1.6 Nitric oxide (NO)

In 1980, Furchgott and Zawadzki published their seminal research on the function of the endothelium in vasomotion. Here it was demonstrated that an intact endothelium is absolutely required for the mediation of ACh-induced vasorelaxation in rabbit aortic rings [91]. These observations defined a role for the endothelium in the production of a vasoactive substance termed endothelial-derived relaxing factor (EDRF) [91] which was subsequently identified as NO [92]. Since then the study of signalling by NO has expanded exponentially and many important physiological roles within the cardiovascular system have been ascribed to this gaseous mediator [93].

NO is synthesised enzymatically by a family of enzymes collectively termed nitric oxide synthases (NOS). The NOS family encompasses three distinct isoenzymes encoded by separate genes that include neuronal NOS (nNOS, NOS-I), inducible NOS (iNOS, NOS-II) and endothelial NOS (eNOS, NOS-III). eNOS is constitutively-expressed within endothelial cells [94] and utilises the amino acid L-arginine as a substrate for the production of NO and L-citrulline under physiological conditions [95]. Numerous agonists have been shown to induce eNOS activation, including ACh, serotonin, PPARs, bradykinin, histamine, Ca^2+^ ionophores, adiponectin, VEGF, fluid shear stress and hypoxia [96], [97]. NOS enzymes comprise an N-terminal oxygenase domain with binding sites for haem, L-arginine and tetrahydrobiopterin (BH_4_), and a reductase domain which binds NADPH, flavin mononucleotide (FMN), FAD, and calmodulin (CaM) (reviewed in [96]). The active eNOS enzyme is a homo-dimer containing a zinc ion, tetrahedrally co-ordinated to cysteine residues at the surface of the dimer [98]. Under quiescent conditions it is anchored to caveolae through its interaction with caveolin-1, and its activation is dependent upon Ca^2+/^CaM, which binds to and displaces eNOS from caveolin, resulting in a conformational change that promotes the movement of electrons from NADPH to the haem moiety in the reductase domain [99]. Its activity can be modulated by numerous posttranslational modifications including phosphorylation at multiple serine, threonine and tyrosine sites [96], palmitylation, glutathionylation, and S-nitrosylation, and association with other proteins such as heat shock protein 90 (Hsp90) [100]. The bioavailability of its substrate, L-arginine and the BH_4_ co-factor are additionally crucial determinants of eNOS function. Indeed, loss of BH_4_ results in eNOS uncoupling and subsequent generation of O_2_^-^ [101].

### 1.7 The role of nitric oxide in angiogenesis

Once formed in the endothelium, NO diffuses to neighbouring SMCs where it activates soluble guanylate cyclase (sGC) by binding to its haem group. sGC converts guanosine triphosphate (GTP) into cyclic guanosine monophosphate (cGMP) which then activates Protein Kinase G (PKG). PKG has a number of downstream targets that act to cause SMC relaxation [102]. The importance of eNOS in controlling vasodilation and blood pressure is highlighted by the vessel-constrictive effects of competitive eNOS inhibitors such as NG-monomethyl-L-arginine (L-NMMA) and NG-nitro-L-arginine-methyl ester (L-NAME) on endothelium-induced vasodilation in *ex vivo* experiments [103], and the clinical use of these inhibitors in the diagnosis of endothelial dysfunction [104]. Direct genetic evidence for eNOS-induced control of blood pressure was confirmed in mice by disruption of the eNOS gene, since these mice displayed enhanced systemic blood pressure compared to wild-type (WT) controls [105]. In addition to its well-established role in regulating vascular tone, NO has also been shown to be a mediator of post-natal angiogenesis (Table 1). Mice in which eNOS has been genetically ablated develop normally, but display a severe form of critical limb ischemia in mouse hindlimb models [106], [107], together with impaired wound healing and defective angiogenesis [108]. Both angiogenic and arteriogenic responses to hindlimb ischemia were severely blunted in eNOS^-/-^ mice, an effect that could be rescued by adenoviral-mediated delivery of a constitutively-active form of eNOS [109].

**Table 1:** A summary of the angiogenic effects of each gasotransmitter and its respective synthetic enzyme, both *in vitro* and *in vivo/ex vivo*. MI: Myocardial Infarction, CORM: CO releasing molecule, L-NAME: NG-nitro-L-arginine-methyl ester, SDF-1: stromal cell-derived factor-1.

**Gasotransmitter**	**Role in angiogenesis (In Vitro)**	**Reference**
**eNOS/NO**	HUVEC form capillary-like structures when stimulated with VEGF, and this is blocked by L-NAME.	[110]
	Overexpression of eNOs, or NO-donors promoted VEGF expression in VSMCs which could act in a paracrine manner to promote endothelial cell proliferation	[125]
**HO/CO**	HO-1 overexpression enhanced endothelial proliferation.	[174]
	HO-1 gene silencing reduced capillary formation, an effect rescued by CORM.	[89]
	CO gas or CORM-2 *inhibited* VEGF-dependent angiogenic responses in HUVECs	[175]
	CORM-2 promoted angiogenic responses in HUVECs, upstream of VEGF	[176]
	Chemical inhibition of HO reduced angiogenic activities of endothelial cells both upstream and downstream of VEGF	[177], [180]
	HO-1 silencing perturbed VEGF-dependent angiogenic responses in endothelial cells	[182]
**CSE/H2S**	Exogenous H_2_S increased HUVEC proliferation.	[90]
	NaHS administration promoted angiogenic responses in cultured endothelial cells	[232]
		
**Gasotransmitter**	**Role in angiogenesis (In Vivo/Ex Vivo)**	**Reference**
**eNOS/NO**	eNOS-/- mice show reduced capillary growth in implanted collagen plugs	[111]
	eNOS-/- mice show impaired angiogenic responses after hindlimb ischemia	[106], [107]
	VEGF-induced angiogenesis is blocked by L-NAME in rabbit cornea	[112]
**HO/CO**	Ablation of HO-1 in mice prevented capillary sprouting in aortic rings in response to SDF-1	[183]
	HO1 inhibition blocks angiogenesis in solid tumours in rats.	[185]
	HO-1 is necessary for post-injury reparative neovascularization after hind-limb ischemia and cutaneous wounding in mice	[187], [188]
**CSE/H**_**2**_**S**	NaHS administration increased neovascularisation and haemoglobin content of Matrigel plugs in mice.	[183]
	NaHS administration promoted angiogenesis after hindlimb ischemia in rats	[233]
	Microvessel formation in aortic rings and wound healing was impaired in CSE^-/-^ mice.	[90]
	NaHS administration improved wound-healing in type-2 diabetic mice	[234]

There is much evidence to support NO-mediating signalling events downstream of VEGF. Thus HUVEC form capillary-like structures when stimulated with VEGF, an effect that can be blocked by the NOS antagonist, L-NAME [110]. *In vivo*, mice with genetic ablation of the eNOS gene (eNOS^-/-^) demonstrated reduced capillary growth in implanted collagen plugs, and impaired angiogenic responses to hindlimb ischemia compared to control animals, and in both cases the phenotype could not be rescued by administration of VEGF [106], [111]. In addition, VEGF-induced angiogenesis was shown to be blocked by L-NAME in a rabbit cornea model of angiogenesis [112]. Mechanistically, the activation of (human) eNOS is associated with phosphorylation at Ser^615^, Tyr^81^, Ser^633^ and Ser^1177^ [113], [114], [115], [116], and has been shown to be mediated by the actions of multiple protein kinases, including Akt/PKB, PKA, c-Src and AMPK [117], [118]. Activation of VEGFR2 in endothelial cells (by VEGF binding) results in the Akt-dependent phosphorylation of eNOS at Ser^1177^ [113], [119], while VEGF treatment of BAECs resulted in the phosphorylation of eNOS at Ser^617^ and Ser^635^ (equivalent to human Ser^615^ and Ser ^633^) [116]. The kinase responsible for phosphorylation of Ser^617^ in this study was suggested also to be Akt, while phosphorylation at Ser^633^ (of human eNOS) has recently been shown to be mediated, in response to VEGF signalling by the serine/threonine-protein kinase, Pim1 [120]. In addition, VEGF-signalling induces the c-Src-dependent phosphorylation and association of Hsp90 with eNOS [121], eNOS is additionally activated by other stimuli, including shear stress [122], which has been shown to be dependent on both Akt- and PKA-dependent mechanisms. NO is believed to promote angiogenesis by a sGC-cGMP-dependent pathway(s), although the precise mechanisms remain to be fully elucidated [123]. There is also evidence that NO can act upstream of VEGF signalling to promote angiogenesis. Thus increased NO, either administered by exogenous NO donors, or due to induction or overexpression of NOS, acted in vascular smooth muscle cells (VSMCs) *in vitro* to increase VEGF expression [124], [125]. In addition, inhibition of NOS *in vivo* eliminated the increased expression of VEGF (and VEGFR-2) in electrically stimulated capillary growth in rat muscle for up to 4 days [126]. Mechanistically, NO has been shown to activate the VEGF promoter *via* the *cis*-regulatory hypoxia response element (HRE) which mediates the transactivation of VEGF transcription by HIF-1 [127]. It should be noted, however, that in an *ex vivo* model of balloon angioplasty in rat thoracic aortae, increased VEGF expression was *inhibited* by NO donors [128] and the reasons for the discrepancies in these observations is currently not clear.

In recent years it has become apparent that asymmetric dimethylarginine (ADMA) and its associated metabolic enzymes dimethylarginine dimethylaminohydrolase I and II (DDAHI and DDAHII) can regulate angiogenesis through their effects on NOS substrate bioavailability [129], [130]. ADMA is an endogenous methylated form of L-arginine that acts as a competitive inhibitor of all NOS enzymes, and is formed from the hydrolysis of proteins that have been methylated on arginine residues. DDAHI and II are enzymes that metabolise ADMA and therefore negate its inhibitory effects on NOS, elevating NO levels [131]. Both *in vitro* and *in vivo* studies have demonstrated that DDAH enzymes are involved in angiogenesis. Aortic rings isolated from DDAHI heterozygous mice (DDAH^+/-^) showed reduced sprouting in Matrigel. This effect could be mimicked through the addition of ADMA to ring explants from WT mice, suggesting that endogenous removal of ADMA by DDAHI is required for vessel growth [132]. Moreover, endothelial-specific ablation of DDAHI blunted angiogenesis both *in vitro* and *in vivo* [133], while gene-targeted over-expression of DDAHI enhanced NO production and angiogenesis in a murine model of hindlimb ischemia that was associated with reduced ADMA levels [134].

### 1.8 Regulation of nitric oxide-dependent signalling by redox mechanisms

The relationship between reactive oxygen species and the signalling functions of NO is complex, and species-type dependent. NO bioavailability is considered to be a fundamental requirement for vascular health [135]. It has long been known that O_2_^-^ readily reacts with NO to form NOO^-^, and so acts to reduce NO bioavailability, thereby contributing to vascular disease aetiology [136]. Indeed, Nox1- and Nox2-derived O_2_^-^ is thought to inactivate NO and enhance hypertension [42], [43], [44], [45] and vascular dysfunction in angiotensin II-induced stress [137]. Nox2 activity has also been shown to directly reduce NO bioavailability leading to enhanced plaque formation in a model of atherosclerosis [138], while detrimental effects of O_2_^-^ on angiogenesis have also been reported [139]. A more oxidising basal cellular redox state can also act to modulate NOS enzymatic activity by direct oxidation of the enzyme itself, *via* glutathionylation [140], [141] or by oxidation of its co-factor, BH_4_ [142]. In both cases this converts the enzyme from a NOS to a superoxide-generating oxidase (or uncoupled NOS), and the NADPH oxidase, Nox2 has been demonstrated to be a potential oxidant source [141], [143]. The responsiveness of the major target of NO, sGC, can also be affected by the cellular redox state. Thus its ferrous haem can become oxidised to an NO-resistant ferric haem which is susceptible to ubiquitylation and degradation [144].

By contrast to O_2_^-^, both exogenous and endogenously-generated H_2_O_2_ have been shown in endothelial cells *in vitro* to promote both NO production and NO-dependent signalling [145] and to stimulate angiogenic responses (proliferation and migration) which are blocked by inhibiting eNOS [146]. Moreover, *in vivo*, the transgenic overexpression of catalase in mice demonstrated that endogenous, endothelial-expressed H_2_O_2_ mediates the upregulation of eNOS after exercise [147]. In BAECs H_2_O_2_ (but not O_2_^-^) was shown to increase both the rate of transcription and the stability of eNOS mRNA in a dose- and time-dependent manner [148]. These increases were shown to be prevented by both the antioxidant N-acetylcysteine (NAC), and the H_2_O_2_ scavenger, catalase, although the precise mechanisms of transcriptional regulation remain unknown. H_2_O_2_ was also shown to be able to upregulate eNOS activity through the modulation of signalling pathways that contribute to its activation *via* VEGF-dependent signalling. Thus Thomas et al. demonstrated that H_2_O_2_ could induce the phosphorylation of eNOS at Ser^1177^, which is known to be necessary and sufficient for VEGF-mediated endothelial cell migration, and this was shown to occur *via* the PI3-K/Akt pathway, shown to be activated by VEGF signalling (see above) [149]. Consistent with this, administration of H_2_O_2_ to BAECs was sufficient to promote NO production *via* Akt and Erk1/2 [150]. Independent of VEGF, H_2_O_2_ has also been shown to be a critical intermediate mediating phosphorylation of eNOS *via* activation of the G-protein-coupled receptor for ADP, P2Y1 [151]. H_2_O_2_ administration has also been shown to stimulate phosphorylation of bovine eNOS at Tyr^83^ (equivalent to Tyr81 in human eNOS) in BAECs *via* Src kinase, although it is unclear whether this is VEGF-dependent or independent [117].

**Fig. 5:**
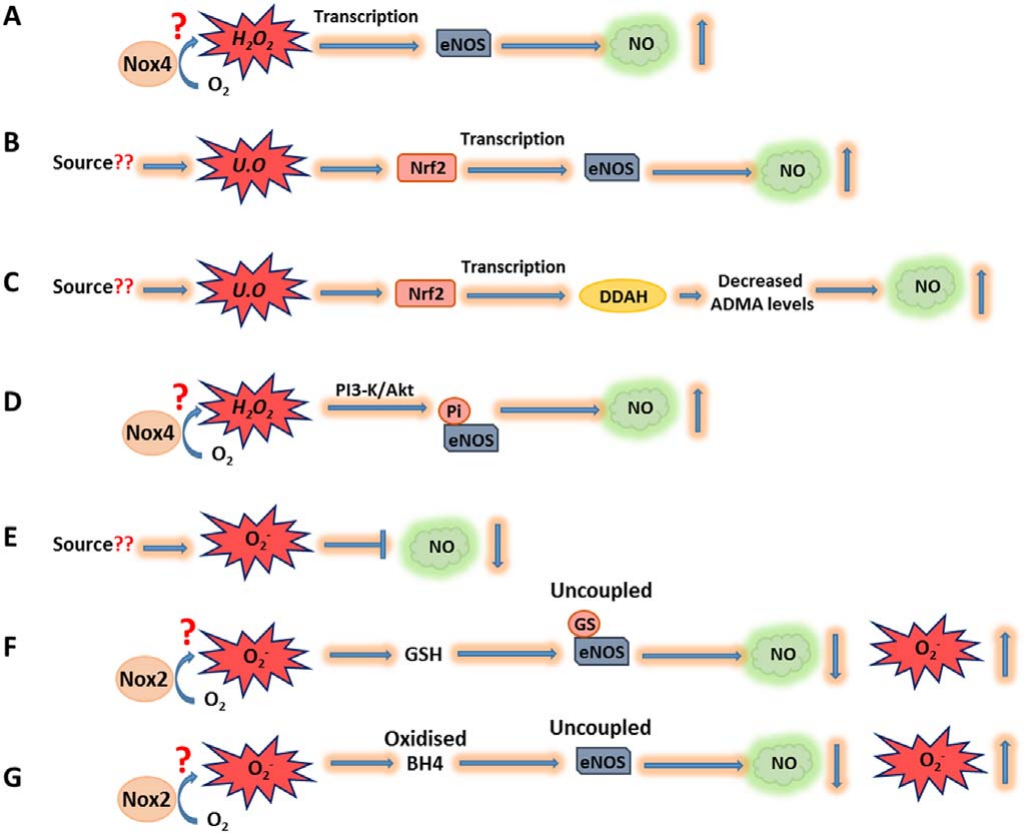
**Reactive oxygen species-dependent mechanisms regulating NO bioavailability. A)** H_2_O_2_ (potentially generated by Nox4) can increase eNOS expression *via* an unknown mechanism leading to increased NO production. **B) & C)** Oxidant-dependent activation of Nrf2 leads to increased transcription of eNOS (and therefore NO production) and DDAH. Increased DDAH expression in turn acts to reduce ADMA (a competitive inhibitor of NOS) levels resulting in increased NO production. **D)** H_2_O_2_ (potentially generated by Nox4) potentiates the activation of the PI3K/Akt signalling pathway which results in phosphorylation and activation of eNOS and increased NO production. **E)** O_2_^-^ is capable of reacting with NO to form peroxynitrite which reduces the bioavailability of NO. **F)** Glutathionylation of eNOS under oxidative conditions and **G)** Oxidation of the eNOS co-factor, BH4, leads to eNOS uncoupling, reduced NO production and O_2_^-^ production. The likely oxidant is O_2_^-^ generated by Nox2. GSH: glutathione, GS: glutathionylation, Pi: phosphorylation, BH_4_: tetrahydrobiopterin, DDAH: dimethylarginine dimethylaminohydrolase, U.O: unidentified oxidant.

As stated previously, H_2_O_2_ is produced endogenously within vascular cells from a number of enzymatic sources including, (perhaps most notably in endothelial cells) Nox4. Indeed, Nox4 overexpression has been shown to increase eNOS protein expression and activity in cultured endothelial cells and to be sufficient to promote proliferation, migration and tube formation [49]. Moreover, *in vivo*, endothelial-specific Nox4-overexpressing mice demonstrated accelerated blood-flow recovery in an ischemic hindlimb model as well as enhanced aortic capillary sprouting. Significantly, these Nox4-induced effects were ablated by genetic deletion of eNOS [49]. Perhaps consistent with these observations, global genetic ablation of Nox4 in mice resulted in an approximately 50% decrease in eNOS expression in the carotid artery, and significantly-reduced NO production which potentially contributed to the attenuated angiogenesis in these mice after femoral artery ligation [46]. Taken together, these studies strongly suggest that Nox4 is a physiological source of enzymatic H_2_O_2_ which acts to promote post-natal angiogenic responses, at least in part, *via* modulation of the expression of eNOS and/or modulation of VEGF-mediated activation of eNOS activity (Fig. 5). In this regard, the potential Nox4-generated H_2_O_2_-dependent oxidative inactivation of protein tyrosine kinases, including PTP1B and SHP2 (negative regulators of VEGFR2 signalling) may be of functional significance [78], [79], [152].

As mentioned above, the bioavailability of NO is also controlled by DDAH enzymes. A recent study has suggested a role for the redox-sensitive transcription factor, Nuclear factor erythroid 2-related factor (Nrf2) in the coordinated regulation of both DDAH and eNOS in renal glomerular endothelial cells [153]. Nrf2 is a basic leucine-zipper transcription factor that is regulated by the cytoplasmic protein Kelch-like ECH-associated protein 1 (KEAP1). In normal physiological conditions, KEAP1 sequesters Nrf2 in the cytoplasm where it is targeted for ubiquitin-mediated degradation. However, upon activation by pro-oxidants (among other stimuli), critical cysteine residues in KEAP1 are oxidised resulting in the liberation of Nrf2 and its subsequent translocation to the nucleus where it drives the expression of a battery of antioxidant and detoxifying genes [154]. The activation of Nrf2 using *tert*-butylhydroquinone was demonstrated to enhance Nrf2 translocation to the nucleus and subsequent DDAHI and II expression [131]. This effect was lost upon Nrf2 ablation. In addition, concomitant with the decrease in ADMA levels observed upon Nrf2 activation, both eNOS expression and NO production increased (Fig. 5). It was concluded that Nrf2 upregulates eNOS as well as DDAH enzymes to potentiate NO production and improve endothelial cell function [153]. The physiologically relevant endogenous source(s) of the oxidant(s) which might activate Nrf2 in this process is not known. However, we have shown that increased Nox4 expression can act to promote the expression of several Nrf2-target genes in other *in vivo* settings (see below) [155], [156], and therefore the increase of Nox4 expression seen in response to hypoxia [49] may be of relevance here.

From these studies it is clear that both O_2_^-^ and H_2_O_2_ play diverse roles in the regulation of NO-dependent signalling, by regulating the expression and activity of eNOS, together with the bioavailability and functional capability of NO itself, and that these regulatory mechanisms have functional consequences in angiogenic phenotypes (Fig. 5).

### 1.9 Carbon monoxide

CO, first denoted as a ‘silent killer’, has come to be recognised as an important physiological mediator in a broad range of biological processes and systems, perhaps most notably in the neurological and cardiovascular fields [157]. CO has high affinity for haemoglobin, and therefore its subsequent ability to reduce O_2_ transport in the blood led to its initial characterisation as a toxic pollutant. However, it later became apparent that CO can be generated physiologically by a family of enzymes known as haem oxygenases (HOs) [158]. This family comprises 3 isoenzymes, HO-1 [159], HO-2 [160] and HO-3 [161], of which only HO-1 and −2 are thought to be catalytically-active proteins. HO-1 is an inducible isoform which has been most closely associated with CO-dependent signalling. Its expression and corresponding activity is both positively and negatively regulated by a number of stimuli including hypoxia [162], [163], heat shock [164] and oxidative stress [165]. It is noteworthy that these stimuli have species-specific effects, with hypoxia upregulating HO-1 in rat tissue [163] but decreasing it in human cells [162]. HO-1 has been shown to be localised to caveolae and cytosolic compartments of endothelial cells [166] as well as the nucleus under conditions of “oxidative stress” [167]. By contrast, HO-2 is constitutively expressed and is responsible for basal CO production primarily in the brain and cardiovascular system [168]. Enzymatically, HO catalyses the rate-limiting step in the breakdown of haem into biliverdin, iron and CO in a reaction that requires oxygen and NADPH [169] Once formed, CO can subsequently induce a number of targeted effects as discussed below.

### 1.10 The role of carbon monoxide in angiogenesis

A number of vascular roles have been ascribed to CO including reduced platelet activation [170] and monocyte-induced inflammation [157]. In addition, CO has been implicated as a vasoactive mediator that can induce vasorelaxation, an effect which has been characterised in a number of vessels including rat aorta [157] as well as mesenteric, renal and pulmonary arteries [171] and has been demonstrated using both CO donors and HO inhibitors [157]. The precise mechanisms by which CO-dependent signalling occurs are not fully understood. However, in common with NO, CO is a non-polar molecule which can freely diffuse across biological membranes and can bind sGC to modulate cGMP production [87]. In addition, some groups have suggested that CO-dependent signalling in the vasculature occurs in an endothelium-dependent manner [172]. Therefore there is likely to be a great deal of interdependence between the vascular functions of CO and NO.

In addition to vascular tone, HO/CO-signalling has also been implicated in angiogenic control (Table 1). HO-1 expression is rapidly induced in bovine aortic endothelial cells in response to hypoxia [173], and early evidence for its functional significance came from studies in which HO-1 overexpression enhanced endothelial cell proliferation [174]. By contrast, targeted gene silencing of HO-1 in endothelial cells reduced capillary formation and cell proliferation. Re-introduction of CO in the form of the CO donors, tricarbonyl-dichlororuthenium (II) dimer (CORM-1) and tricarbonylchloro(glucinato)ruthenium (II) (CORM-3), was sufficient to recover the angiogenic properties of HO-1 deficient endothelial cells [89]. CO-dependent signalling has been reported to act both upstream and downstream of VEGF induction in the angiogenetic response, although it should be noted that there remain inconsistencies within the literature with regard to its pro- or anti-angiogenic effects [175], [176], [177]. This may reflect the differences in cell types and/or differences in levels of CO applied to the cells [178].

Upstream of VEGF-signalling, HO-1 overexpression or activation has been shown to increase VEGF production in VSMCs, macrophages and endothelial cells [177], [179] and accordingly treatment of endothelial cells with CORM was shown to mimic this effect [177]. Consistent with this, inhibition of HO activity by tin protoporphyrin IX (SnPPIX) abrogated the increase in VEGF expression in VSMCs, observed upon stimulation by hypoxia, IL-1β or hemin [180], while ablation of HO-1 was shown to inhibit VEGF expression and the angiogenic responses of endothelial cells [89]. By contrast, in a separate study, the hypoxic induction of VEGF in rat aortic smooth muscle cells was shown to be supressed by both CO and NO [181] in a sGC- and cGMP-mediated pathway that resulted in decreased HIF-1 binding to the VEGF enhancer. Downstream of VEGF signalling, CO has also been reported to promote both pro- and anti-angiogenic effects. Thus VEGF was shown to induce sustained HO-1 expression and activity in cultured endothelial cells, and the angiogenic responses induced by VEGF were perturbed when HO-1 was inhibited [182]. However, in a more recent study using HUVEC, exposure to CO was found to inhibit VEGF-induced angiogenic responses [175]. Mechanistically, CO administration acted to suppress VEGF-stimulated VEGFR-2 phosphorylation and the downstream Akt phosphorylation. The discrepancies in these observations are likely to be due to differences in experimental design and remain to be reconciled. HO-1 has also been shown to mediate the pro-angiogenic effects of SDF-1, through a protein kinase C-ζ-dependent, VEGF-independent mechanism. Thus SDF-1 was shown to induce HO-1 expression in endothelial cells, and ablation of HO-1 in mice, *in vivo*, prevented aortic rings forming capillary sprouts in response to SDF-1 [183]. Mechanistically, SDF-1 was shown to promote the phosphorylation of vasodilator-stimulated phosphoprotein (VASP) *via* HO-1 activation to promote angiogenesis. In addition to modulating the expression and associated signalling pathways of pro-angiogenic factors, HO-1 has also been shown to act to deplete the levels of the anti-angiogenic factors, soluble fms-like tyrosine kinase I and soluble Endoglin, thereby potentially further enhancing angiogenesis [184].

*In vivo*, a potential pro-angiogenic role of HO/CO-dependent signalling has also been demonstrated. Thus the pan HO inhibitor, zinc protoporphyrin (ZnPP) was found to block angiogenesis in solid tumours suggesting a physiological role for HO in neovascularisation [185]. In addition, the induction of HO-1 activity, or the administration of a CO-donor both acted to promote formation of new coronary arteries after myocardial infarction induced by coronary artery ligation in rats [186]. In addition HO-1 was shown to be necessary for efficient post-injury reparative neovascularization after both hind-limb ischemia [187] and cutaneous wounding in mice [188]. A critical role for HO-1 placental vascular formation during embryonic development is also recognised. Thus HO-1 expression and endogenous CO production is highly elevated in the placenta during pregnancy [189] and a partial deficiency of maternal HO-1 results (in mice) in the restriction of the growth of both the placenta and the foetus that is due, at least in part, to impaired angiogenesis [190].

The manipulation of the HO/CO axis using pharmacological and genetic means has therefore implicated this pathway in the regulation of angiogenesis. However little is known about the exact mechanism(s) through which CO acts. As stated above, CO is believed to induce changes in protein activity primarily through its reaction with haem containing proteins [191], and both sGC and NOS have been implicated as potential targets, again suggesting cross-talk between gasotransmitters [192].

### 1.11 Regulation of carbon monoxide-dependent signalling by redox mechanisms

It is well-established that pro-oxidant-generating stimuli such as UVA radiation and cadmium chloride, in addition to H_2_O_2_ *per se*, are capable of inducing HO gene expression [193]. Indeed, HO-1 expression has been used as a model system for studying redox-regulated gene expression in mammalian cells [194]. Administration of H_2_O_2_ has been demonstrated to enhance both HO-1 and VEGF expression in a variety of cell types, including vascular cells, while chemical inhibition of HO-1 activity in part abrogated the increase in VEGF expression, underlying the significance of this redox-regulation to angiogenesis [195]. From several studies it has become apparent that many redox-sensitive cell signalling pathways and transcription factors are involved in regulating HO-1 expression including Mitogen Activated Protein Kinases (MAPKs) such as P38 [196] and JNK [197] as well as the transcription factor Hypoxia Inducible Factor 1α (HIF1α) [163] and, perhaps most notably, Nrf2 [156]. Cigarette smoke contains a number of pro-oxidants and application of aqueous extracts to NIH3T3 cells induced Nrf2 activation leading to HO-1 induction, an effect that was lost when Nrf2 was silenced [198]. Nrf2 has been shown to induce HO-1 transcription through two distal *cis*-acting enhancer elements in the HO-1 promoter that both contain Nrf2-binding consensus antioxidant response elements (AREs) [198]. The regulation of HO-1 in response to oxidants is further complicated by the involvement of a hypoxia-induced repressor of HO-1 transcription termed BTB Domain and CNC Homolog 1 (Bach1) [199]. By contrast to Nrf2, the activity of Bach1 is inactivated by oxidants and it was shown in human keratinocytes that arsenite-mediated HO-1 induction involves both the removal of Bach1 from the HO-1 promoter and the subsequent Nrf2-directed up-regulation in its expression [200]. Indeed, hypoxia-dependent suppression of HO-1 in human endothelial cells appears to be regulated by a complex interplay between Bach1 and Nrf2 [162], which is in turn potentially mediated by redox-dependent mechanisms.

**Fig. 6:**
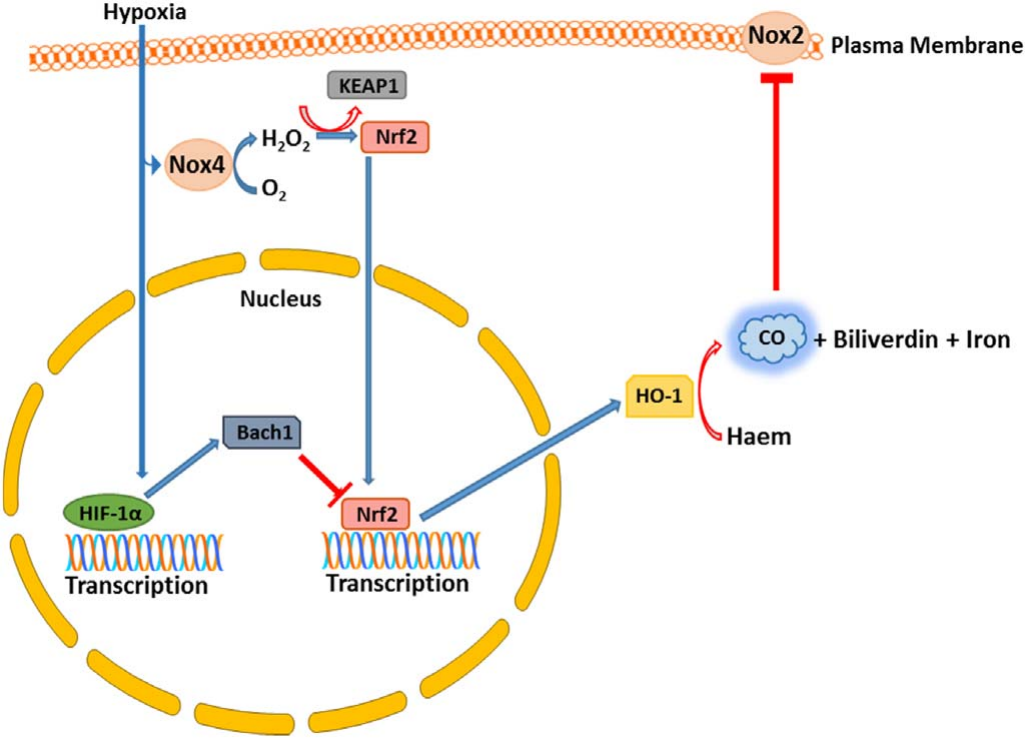
**The inter-relationships between hypoxia, NADPH oxidases and CO.** Hypoxia can act to increase Nox4 expression and H_2_O_2_ production, potentially leading to Nrf2 activation and increased HO-1 expression. In opposition to this, hypoxia also increases the expression of Bach1, a negative regulator of Nrf2 function and HO-1 expression. This interplay between transcription factors may therefore modulate CO production by HO-1. CO can itself also inhibit the activity of Nox2.

Although it is well accepted that the Nrf2/HO-1 response is (in part) oxidant driven, the physiological endogenous source(s) of these oxidants remains unknown. However we have shown that Nox4-generated H_2_O_2_ can activate Nrf2 and induce HO-1 expression in cardiac-specific Nox4-overexpressing hearts [155]. Moreover, *in vitro* simulation of hemodynamic stress, using phenylephrine-stimulated neonatal rat ventricular myocytes, resulted in an increase in both Nox4 and Nrf2 protein expression, and a subsequent up-regulation in HO-1 mRNA expression. This effect was ablated when either Nox4 or Nrf2 were silenced [156] (Fig. 6). Consistent with these observations, global Nox4-null mice display reduced HO-1 expression in isolated lung endothelial cells, an effect that resulted in enhanced apoptosis. This has been attributed to perturbed CO production as re-introduction of CO using CORMs enhanced lung endothelial cell survival [46].

Intriguingly, there is also potential for the converse cross talk between CO- and Nox-dependent signalling mechanisms. Thus it has been demonstrated that CO administration can act to inhibit the activity of Nox2 within human airway smooth muscle cells and neutrophils [201] due to binding to the haem moiety. It will be of interest to determine whether this is a common regulatory mechanism, potentially modulating the activity of other NADPH oxidases.

### 1.12 Hydrogen sulphide

H_2_S was considered a toxic environmental pollutant until Abe and Kimura first proposed it to be an endogenous cellular signalling mediator [202]. Subsequent extensive research efforts have established H_2_S as a third member of the gasotransmitter family of signalling molecules and have ascribed to it a number of diverse functions within different physiological systems including the cardiovascular, immunological and nervous systems [203]. H_2_S is synthesised endogenously through the action of 3 pyridoxal 5′ phosphate- (vitamin B6)-dependent enzymes, cystathionine β-synthase (CBS), CSE and the combined action of 3-mercaptopyruvate sulphur transferase (3MST) and cysteine aminotransferase (CAT) [204]. Each of these enzyme systems displays promiscuity with respect to substrate specificity and function, and can additionally catalyse other reactions in the sulphur metabolic network [205]. These enzymes also display tissue-specific patterns of expression. Thus CBS plays a major role in H_2_S generation in the nervous system [206], while 3MST is widely distributed in multiple cell types including neurons, hepatocytes, cardiac cells and endothelial cells [207], [208]. However, within the vascular system, the major enzymatic source of H_2_S is CSE, which has been shown to be expressed and be active in a number of vascular cell types including SMCs [209], perivascular adipose cells [210] and endothelial cells [211], [212], [213]. CSE is a tetrameric protein and it generates H_2_S from a number of substrates including cysteine, cystine and homocysteine [205], [214]. Subcellularly CSE and CBS have been found in the cytoplasm [215], but have also been suggested to be in the nucleus [216] and under hypoxic conditions in the mitochondria [217], [218]. CAT and 3MST are generally considered to be localised to the mitochondria (see Fig. 4) [219], [220].

The chemical properties of H_2_S are such that under physiological conditions in aqueous solutions at pH 7.4, H_2_S exists in a dissociated state with approximately two thirds of total H_2_S existing as hydrosulphide anions (HS^-^) and protons (H^+^) with the remaining third being in the form of H_2_S undissociated, (dissolved) gas. HS^-^ can subsequently form sulphide ions (S_2_^-^) through further dissociation but only at high, non-physiological pH. [S_2_^-^] is therefore considered negligible in physiological systems [221]. Undissociated H_2_S is a non-polar, lipophilic molecule which can diffuse through membranes in a similar manner to NO and CO. However, due to its dissociation at physiological pH, H_2_S should be considered to be less lipid- permeable than the other gasotransmitter molecules [222], and this may impact on its signalling functions. The term H_2_S is typically used collectively to denote both H_2_S and HS^-^ anions as it is currently unknown which of these is the most physiologically relevant species. Once generated, H_2_S is believed to function as a secondary messenger by directly modulating the function of downstream targets. In common with both NO and CO, H_2_S can form coordination complexes with metal centres (such as haem groups) within proteins [223]. In addition, it acts to covalently modify cysteine residues on target proteins in a process known as S-sulfhydration [224]. Here, sulphur derived from H_2_S becomes added to the thiol group of a target cysteine residue to render the formation of a hydropersulphide moiety (-SSH). In one study it was shown that as much as 10–25% of some abundant proteins in mouse liver including actin, tubulin and glyceraldehyde-3-phosphate (GAPDH) exist in the S-sulfhydration state under physiological conditions [224]. Further, this was shown to be mediated by H_2_S generated enzymatically from L-cysteine by CSE. Thus the limited number of specific H_2_S target proteins currently identified [224] seems likely to grow.

### 1.13 The role of hydrogen sulphide in angiogenesis

One of the first biological activities attributed to H_2_S was in the regulation of vascular tone [225]. Subsequent research efforts using a combination of H_2_S donors [210], [226], [227], [228], [229] and direct genetic manipulation of the CSE gene have confirmed the physiological relevance of the CSE/H_2_S pathway in vasorelaxation and blood pressure regulation [230]. At the cellular level, studies using patch clamp techniques have demonstrated that both endogenous and exogenous H_2_S can directly hyperpolarize resistance artery VSMCs *via* modulation of ATP-gated K^+^(K_ATP_) channel activity, and that these effects are independent of cGMP-mediated phosphorylation [231]. More recently, H_2_S has been demonstrated to be an endothelial-derived hyperpolarizing factor (EDHF) which exerts its vasodilatory actions on VSMCs by causing the opening of K_ATP_ channels, at least in part, through S-Sulfhydration of the Kir 6.1 subunit at cysteine-43 [212]. In addition to its actions as an EDHF acting on adjacent smooth muscle, H_2_S signalling is also thought to be important within endothelial cells themselves. Endothelial CSE can be activated by Ca^2+^/calmodulin [230] in response to cholinergic stimulation or by the calcium ionophore, A23187 leading to enhanced H_2_S production [230]. Moreover, stimulation of isolated endothelial cells with ACh resulted in hyperpolarisation in cells isolated from WT but not CSE^-/-^ mice. This effect is mediated not by K_ATP_ channels but by small and intermediate calcium-gated K^+^ channels present within the endothelium, as the hyperpolarisation could be blocked by apamin and charybdotoxin [212]. Whether specific cysteine residues within these calcium-gated K^+^ channels are targets of S-Sulfhydration remains to be demonstrated.

H_2_S has also been shown to be a key regulator of angiogenesis (Table 1). Thus topical application of H_2_S to endothelial cells *in vitro* increased HUVEC proliferation as well as migration and capillary morphogenesis on Matrigel, which are considered essential initiating steps in the angiogenic response [90], [232]. *In vivo*, administration of NaHS (an H_2_S donor) increased neovascularisation and haemoglobin content of Matrigel plugs in mice [232] and, perhaps consistent with this, also led to improved capillary sprouting and blood flow recovery in a rat hindlimb ischemia model [233]. The significance of CSE-derived H_2_S has also been demonstrated *in vivo*, since both microvessel formation in response to VEGF and wound healing were impaired in CSE^-/-^ mice compared to wild-type littermates [90]. In a separate study H_2_S was also shown to improve wound healing in type 2 diabetic mice *via* the activation of angiopoietin-1 [234]. Clinically, low plasma levels of H_2_S and reduced placental CSE expression are associated with women with pre-eclampsia and it has therefore been suggested that CSE may have important roles in placenta vascularisation [213].

Mechanistically, several studies have demonstrated that the proangiogenic effects of H_2_S involve the activation of both P13K-Akt and MAPK signalling pathways [90], [211], [232]. In this regard it might be significant that Phosphatase and Tensin Homolog (PTEN), a negative regulator of Akt-signalling has been proposed to be a potential direct target of H_2_S-mediated modification [235]. There is also increasing evidence to support the involvement of H_2_S-mediated modification of K_ATP_ channels in the stimulation of the MAPK signalling pathway(s). Thus it has been shown that the activation of K_ATP_ channels *per se* is sufficient to promote angiogenesis in endothelial cells *in vitro* [236] and conversely that K_ATP_ inhibitors abrogate VEGF-dependent angiogenic responses. Moreover, in one study the H_2_S-dependent activation of p38 MAPK was shown to occur *via* a K_ATP_ channel-dependent mechanism [90], that was shown to act downstream of VEGF-signalling. In addition, NaHS administration to tumour-derived human EC cells resulted in a proangiogenic cellular phenotype that was characterised by increased K^+^ and nonselective cationic currents, and increased cytosolic calcium [237]. These effects were again shown to act downstream of VEGF-signalling. CSE was identified in both these studies as the relevant endogenous source of H_2_S, as genetic [90] or pharmacological [237] ablation of CSE activity in both cases ablated the pro-angiogenic effects of VEGF. Another potential mechanism of action of H_2_S in promoting angiogenesis might be *via* the modulation of cGMP levels. As stated above, the importance of sGC/cGMP-dependent signalling (downstream of VEGF) in angiogenesis has been demonstrated previously. H_2_S has been shown to reduce the sGC haem-Fe from a ferric to a ferrous state, thereby promoting cGMP production [238]. In addition, H_2_S is an inhibitor of phosphodiesterase (PDE) activity (which breaks down cGMP), and therefore can further promote cGMP levels and signalling functions [239].

Recently the potential roles of miRNAs in the mediation of the proangiogenic effects of H_2_S were investigated. miR-640 was identified as an miRNA which was significantly downregulated in vascular ECs by H_2_S treatment and conversely overexpression of miR-640 reduced the proangiogenic effects of H_2_S [240]. The altered expression of miR-640 in response to H_2_S was blocked by inhibition of either VEGFR2 or mTOR, again suggesting the involvement of VEGF- and Akt-dependent signalling in the angiogenic response. However, mechanistically, miR-640 was shown to be a negative regulator of HIF1α, *via* direct binding to 3′UTR sequences within HIF1α mRNA. Perhaps consistent with these observations, the loss of CSE-derived H_2_S has been shown to be associated with decreased HIF-1α [241]. Thus H_2_S may potentially act upstream as well as downstream of VEGF-signalling to promote angiogenic responses and a feed-forward cycle between H_2_S production and HIF-signalling has been proposed [242]. CSE-derived H_2_S production has also been implicated in the negative regulation of expression of anti-angiogenic factors. For instance, CSE silencing in HUVEC resulted in the increased release of soluble fms-like tyrosine kinase I as well as soluble endoglin [213], while over-expression of CSE conversely dampened their release. Moreover, administration of the slow-releasing H_2_S donor, GYY4137, caused a reduction in circulating levels of these anti-angiogenic factors. Therefore H_2_S may potentially act upon multiple (potentially inter-related) signalling pathways to modulate the cellular angiogenic response.

### 1.14 Regulation of hydrogen sulphide-signalling by redox-dependent mechanisms

As is the case for NO and CO, the signalling functions of H_2_S that involve binding to metal centres can be modulated by the redox state of the coordinated metal ion, which in turn may be modulated by both the “steady state” redox potential of the cellular environment and/or the localised production of reactive oxygen species such as O_2_^-^ or H_2_O_2_. In addition, the signalling mechanisms of H_2_S which involve thiol modification may also be subject to redox-dependent regulation. The precise mechanism through which H_2_S induces its modifications remains contentious. It was thought initially that H_2_S attacks previously oxidised cysteine residues such as those in the sulphenic acid (SOH) or disulphide bonded (S-S) state in a reduction reaction [224]. However recent studies have suggested that in order to mediate its function, H_2_S acts as an oxidant molecule through an initial reaction with H_2_O_2_ to form polysulphides (H_2_S_n_). These polysulphides can then oxidise cysteine residues on target proteins yielding the persulphide moiety [226], [235]. Such a mechanism of H_2_S-mediated oxidative activation, ultimately resulting in the formation of an intermolecular disulphide bond, has been demonstrated in the case of PKG [226]. Moreover, the H_2_O_2_-dependent conversion of H_2_S to polysulphides results in the loss of polarity, and therefore greater lipid solubility of the signalling moiety. This therefore potentially increases the ability of H_2_S to signal to adjacent cells, and may be an important contributory factor in regulating the signalling functions of H_2_S.

**Fig. 7:**
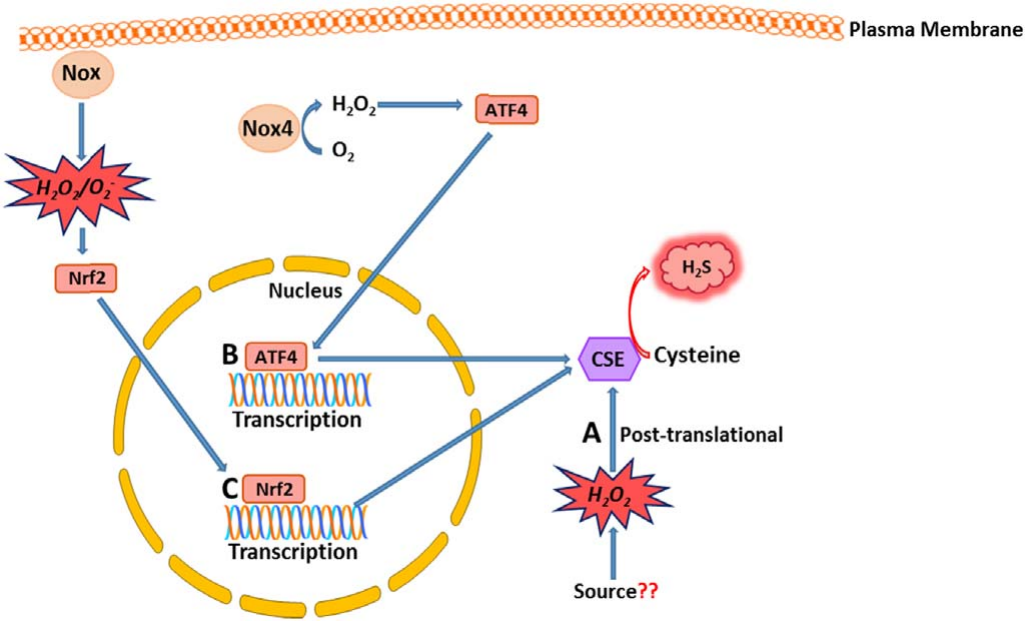
**Redox-dependent mechanisms regulating H**_**2**_**S production by CSE. A)** H_2_O_2_ (generated from an unknown source) can enhance CSE activity. **B)** Nox4 represents a potential source of H_2_O_2_ that can upregulate the transcriptional expression of CSE through an ATF4-dependent mechanism. **C)** NADPH-derived O_2_^-^/H_2_O_2_ can activate Nrf2 leading to increased CSE transcription. These redox-dependent transcriptional and post-translational changes in CSE expression may lead to altered H_2_S production. CSE: Cystathionine-γ-lyase.

Another potential level at which reactive oxygen species may act to mediate H_2_S-dependent signalling is by regulation of CSE-dependent H_2_S production. At the level of gene expression, Wang et al. showed that exogenous H_2_O_2_ increased the activity of the CSE promoter [243] while in another study CSE transcription was upregulated in rat mesangial cells by treatment with platelet-derived growth factor, and this upregulation was abolished by antioxidants including NAC and the Nox inhibitor, DPI [244]. However in the interpretation of these results it should be noted that DPI is an inhibitor of *all* flavoproteins, including NOS [245]. A potential role for Nox4 in the transcriptional regulation of CSE is also suggested by studies conducted in our group. Thus we have demonstrated that in endothelial cells *in vitro*, Nox4-generated H_2_O_2_ acts as a positive regulator of CSE transcription*, via* activation of a haem-regulated inhibitor kinase/ eIF2α/activating transcription factor 4 (ATF4) signalling module and CSE was found to be a direct transcriptional target of ATF4 [246]. *In vivo*, endothelial-specific Nox4 transgenic mice exhibited lowered blood pressure [48] and a hypo-contractile phenotype in response to phenylephrine that was abolished when vessels were incubated with the CSE inhibitor, propargylglycine [246]. We therefore conclude that Nox4 may be a physiologically relevant source of H_2_O_2_ that positively regulates CSE expression and H_2_S production [246] in endothelial cells (Fig. 7).

In addition to playing a role in the transcriptional regulation of CSE, H_2_O_2_-dependent signalling may also modulate CSE activity. Thus in HUVECs it was shown, using a cell-trappable fluorescent H_2_S probe, that VEGF application acted to increase CSE-derived H_2_S production in a H_2_O_2_- dependent manner. The relevant source of H_2_O_2_ in these experiments was again suggested to be a Nox, as the increase in H_2_S production could be inhibited by DPI (although, as stated above, DPI is not a specific Nox inhibitor). Clearly, however, crosstalk between H_2_S and H_2_O_2_, in endothelial cells after VEGF-activation, may be important in the regulation of angiogenic cellular responses [247].

### 1.15 Interactions between gasotransmitters

The sequential discovery and characterisation of each of the gasotransmitters had initially led them to be considered as biologically distinct entities that mediate their functions independently of each other. While this is no doubt the case in certain settings, it has become apparent that considerable synergistic and antagonistic interplay occurs between this family of gases at multiple levels creating a multifaceted signalling platform though which key cellular and physiological processes, such as angiogenesis, are orchestrated [87]. Coletta et al. studied the synergistic effects of H_2_S and NO in the angiogenic response. Here, the pro-angiogenic effects of H_2_S were ablated in endothelial cells isolated from eNOS gene-deficient mice. Conversely the silencing of CSE and concomitant reduction in H_2_S production prevented NO-stimulated angiogenesis. Mechanistically, the mutual dependency between H_2_S and NO in the endothelium occurs on multiple levels and appears to be centred on the production of cGMP. NO is known to bind to and activate sGC leading to elevated cGMP production. By contrast H_2_S inhibits phosphodiesterase-5 (PDE5) preventing cGMP degradation. Furthermore H_2_S was also shown to enhance NO production in endothelial cells. Thus these gasotransmitters act in synergy to potentiate the effect of cGMP leading to protein kinase G (PKG) activation and subsequent modulation of key aspects of angiogenesis [123]. Interestingly, the haem group of sGC can be bound and activated by CO and NO but with varying affinities. CO, for example, binds at lower affinity than NO, an observation that may relate to compensatory actions of the gases [248]. By contrast H_2_S can reduce the haem iron of sGC from its ferric form to its ferrous form, an effect that promotes the binding of NO [238]. This ability for a single protein to become the target of multiple gasotransmitters has been shown to extend to a number of other proteins such as GAPDH which can be S-sulfhydrated by H_2_S and nitrosolated by NO [224].

The interrelationship between NO and CO extends further as it has been shown that NO can stimulate HO in the endothelium [249] but inhibit it in the brain [250]. To add a further layer of complexity to the interplay between the gases, it is becoming apparent that they may be able to react together to form novel signalling intermediates. Indeed the NO donor sodium nitroprusside (SNP) and H_2_S can react to form nitroxyl (HNO) [251] which has been shown to elicit anti-angiogenic effects in tumours [252]. Taken together it is clear that the effects of these gases cannot be viewed simply in terms of their individual functions and must be considered as part of a more complex system, in which many aspects of their biology are crucial, including, for example, their subcellular location. It is conceivable that compartmentalisation of the gasotransmitter producing enzymes into the same subcellular location may facilitate the interaction of their respective gases thereby helping to fine-tune the varied signalling responses associated with them. Perhaps consistent with this notion, O_2_^-^ and H_2_O_2_ (enzymatically produced in a highly-regulated, compartmentalised manner), can potentially act in a similar way through varied and complex interactions with the gasotransmitters to yield diverse signalling molecules such as polysulphide [226] and peroxynitrite [253]. A greater understanding of the chemistries and interrelated actions of these gases is now very much needed and will no doubt lead to a fuller understanding of their roles in many biological processes, including angiogenesis.

## 2 Conclusion and future perspectives

In the last decade the conventional paradigm of reactive oxygen species as deleterious by-products of aerobic metabolism has shifted, and now it is largely accepted that they represent a group of highly regulated and coordinated secondary messengers capable of mediating a number of physiological processes [254] including angiogenesis. In particular, H_2_O_2_ has been shown to induce endothelial cell phenotypes such as increased proliferation, tube formation and migration that are all fundamental to the angiogenic response [52]. Nox enzyme complexes have emerged as the significant endogenous sources of O_2_^-^ and H_2_O_2_ within the vasculature and Nox4, in particular, seems a likely generator of the H_2_O_2_ shown to be important in the angiogenic cellular response. Thus Nox4 is highly expressed in vascular cells compared to other Nox isoforms, and its expression increases in response to tissue hypoxia [67], a condition in which many pro-angiogenic factors such as VEGF are released. Secondly, by contrast to Nox1 and Nox2 which generate O_2_^-^, Nox4 is considered to be a generator of H_2_O_2_, the oxidant species shown to be most pro-angiogenic [53]. Lastly, genetic gain- and loss-of-function models, both *in vitro* and *in vivo,* have consistently highlighted a positive role for Nox4 in ischemia-induced angiogenesis [46], [49].

The roles of reactive oxygen species, notably Nox-generated O_2_^-^ and H_2_O_2_, in orchestrating the angiogenic cellular responses are being demonstrated to be increasingly complex and diverse. Moreover it is clear that their functions are intimately connected to those of the gastransmitters, NO, CO and H_2_S. The production of all these reactive signalling molecules occurs in a highly coordinated manner, close to their biological sites of action. They can also act independently, synergistically or antagonistically to elicit their downstream effects often culminating in a highly fine-tuned response. The transient chemical nature of the gasotransmitters, as well as their inherent need to be produced at their sites of action, and their toxicity in non-physiological concentrations, precludes their direct use in modulating angiogenesis therapeutically. However, the potential use of (sometimes slow-releasing) donor compounds of NO, CO and H_2_S as clinical therapeutics is being vigourously explored [255]. In this regard a critical issue is the adverse, off target, potential pro-oncogenic effects which might be induced by promoting angiogenesis. Thus it will be necessary to specifically target the activity of such drugs, and a better understanding of the complexity of the interplay within and between different reactive oxygen species and the gasotransmitters is crucial. This represents a major challenge to the scientific community, and better methodologies to measure the physiological (and pathophysiological) levels of these mediators accurately and efficiently both *in vitro* and *in vivo* are in high demand. Interestingly, donor molecules have begun to be designed which exploit the beneficial synergistic actions of the gasotransmitters. One such molecule; ZYZ-803, is a novel H_2_S- NO hybrid molecule which has been shown to exhibit greater proangiogenic potency than either H_2_S or NO alone [256]. It can be hoped that in the future a more integrative approach to studying the roles of the gasotransmitters in angiogenesis, and their modes of regulation by diverse redox-dependent mechanisms may lead to the development of more efficacious therapeutic strategies.

## Conflict of interest statement

None.
